# Curative or pre-emptive adenovirus-specific T cell transfer from matched unrelated or third party haploidentical donors after HSCT, including UCB transplantations: a successful phase I/II multicenter clinical trial

**DOI:** 10.1186/s13045-017-0469-0

**Published:** 2017-05-08

**Authors:** Chongsheng Qian, Arnaud Campidelli, Yingying Wang, Huili Cai, Véronique Venard, Hélène Jeulin, Jean Hugues Dalle, Cécile Pochon, Maud D’aveni, Benedicte Bruno, Catherine Paillard, Stéphane Vigouroux, Charlotte Jubert, Patrice Ceballos, Aude Marie-Cardine, Claire Galambrun, Clément Cholle, Isabelle Clerc Urmes, Nadine Petitpain, Marcelo De Carvalho Bittencourt, Véronique Decot, Loïc Reppel, Alexandra Salmon, Laurence Clement, Danièle Bensoussan

**Affiliations:** 1 Unité de Thérapie cellulaire et Tissus and FR 3209, CHRU de Nancy, Vandoeuvre-Lès-Nancy, F54511 France; 2 UMR 7365 and FR 3209 CNRS-UL-CHU, Université de Lorraine, Vandoeuvre-Lès-Nancy, F54511 France; 3 Laboratoire d’Immunologie and Plateforme Nancytomique, CHRU de Nancy, Vandoeuvre-Lès-Nancy, F54511 France; 4 Laboratoire de Virologie, CHRU de Nancy, Vandoeuvre-Lès-Nancy, F54511 France; 5 Immuno-Hématologie pédiatrique, Hôpital Robert Debré, Paris, F75935 France; 6 Hématologie pédiatrique, Hôpital Jeanne de Flandres CHU de Lille, Lille cedex, F59037 France; 7 Pédiatrie III, Hôpital de Hautepierre, Strasbourg, F67098 France; 8 Groupe hospitalier Sud Hôpital Haut-Lévêque, Hématologie clinique et thérapie cellulaire, Pessac Cedex, F33604 France; 9 Hématologie Oncologie Pédiatrique, Hôpital des Enfants Pellegrin, Bordeaux, F33000 France; 10 Hématologie Clinique, Hôpital St Eloi, Montpellier, Cedex 5 F34295 France; 11 Hématologie et Oncologie Pédiatrique, Hôpital Charles Nicolle-CHU de Rouen, Rouen, F76031 France; 12 Immuno-hématologie Pédiatrique, CHU de la Timone, Marseille, F13385 France; 13 Unité de Transplantation Médullaire Allogénique, CHRU de Nancy, Vandoeuvre-lès-Nancy, F54511 France; 14 Faculté de Pharmacie, Département de Microbiologie-Immunologie, Université de Lorraine, Nancy, F54001 France; 15 Plateform of Clinical Research Facility PARC, Unit of Methodology, Data Management and Statistics, CHRU de Nancy, Vandoeuvre-Lès-Nancy, F54511 France; 16 Centre Régional de Pharmacovigilance de Lorraine, CHRU de Nancy, Vandoeuvre-Lès-Nancy, F54511 France

**Keywords:** Adenovirus-specific T cells, Interferon-γ-based immunomagnetic isolation, Allogeneic stem cell transplantation, Umbilical cord blood transplantation, Third party haploidentical donor

## Abstract

**Background:**

Allogeneic hematopoietic stem cell transplantation (HSCT), the most widely used potentially curable cellular immunotherapeutic approach in the treatment of hematological malignancies, is limited by life-threatening complications: graft versus host disease (GVHD) and infections especially viral infections refractory to antiviral drugs. Adoptive transfer of virus-specific T cells is becoming an alternative treatment for infections following HSCT. We report here the results of a phase I/II multicenter study which includes a series of adenovirus-specific T cell (ADV-VST) infusion either from the HSCT donor or from a third party haploidentical donor for patients transplanted with umbilical cord blood (UCB).

**Methods:**

Fourteen patients were eligible and 11 patients received infusions of ADV-VST generated by interferon (IFN)-γ-based immunomagnetic isolation from a leukapheresis from their original donor (42.9%) or a third party haploidentical donor (57.1%). One patient resolved ADV infection before infusion, and ADV-VST could not reach release or infusion criteria for two patients. Two patients received cellular immunotherapy alone without antiviral drugs as a pre-emptive treatment.

**Results:**

One patient with adenovirus infection and ten with adenovirus disease were infused with ADV-VST (mean 5.83 ± 8.23 × 10^3^ CD3+IFN-γ+ cells/kg) up to 9 months after transplantation. The 11 patients showed in vivo expansion of specific T cells up to 60 days post-infusion, associated with adenovirus load clearance in ten of the patients (91%). Neither de novo GVHD nor side effects were observed during the first month post-infusion, but GVHD reactivations occurred in three patients, irrespective of the type of leukapheresis donor. For two of these patients, GVHD reactivation was controlled by immunosuppressive treatment. Four patients died during follow-up, one due to refractory ADV disease.

**Conclusions:**

Adoptive transfer of rapidly isolated ADV-VST is an effective therapeutic option for achieving in vivo expansion of specific T cells and clearance of viral load, even as a pre-emptive treatment. Our study highlights that third party haploidentical donors are of great interest for ADV-VST generation in the context of UCB transplantation. (N° Clinical trial.gov: NCT02851576, retrospectively registered).

**Electronic supplementary material:**

The online version of this article (doi:10.1186/s13045-017-0469-0) contains supplementary material, which is available to authorized users.

## Background

Allogeneic hematopoietic stem cell transplantation (HSCT) is a curative option for treatment of some hematological diseases, malignant and non-malignant. Although improvements have been performed in recent years, there remains a risk of opportunistic infections in a context of severe immunodeficiency especially in HSCT with human leukocyte antigen (HLA) mismatched or matched unrelated donors ((M)-MUD), umbilical cord blood (UCB), or haploidentical donors [1–5].

Among infections, viral reactivations such as adenovirus (ADV), cytomegalovirus (CMV), BK virus, and Epstein-Barr virus (EBV), worsening in post-transplant lymphoproliferative disease (PTLD), are associated with high morbidity and mortality, especially after alternative HSCT [3, 4], mainly due to impaired specific immune reconstitution [5–7]. The incidence of fatal viral infection is 17–20% with unrelated donors [8]. In children, ADV infection and disease are the most common infectious complications, with reported incidence varying from 6 to 28% post-HSCT [9–11]. Adults may also be affected, although less frequently from 0 to 6% [9, 12]. Progression to disseminated ADV disease occurs in 10–20% of infected patients and is associated with a high mortality rate (20–80%) [10, 13, 14]. However, the incidence of ADV systemic infection varies dramatically according to recipient’s age [9].

Pre-emptive antiviral treatment before the appearance of clinical signs of viral disease, due to regular monitoring of viral load, has improved survival [10, 15]. An antiviral drug, cidofovir, seems to be effective in the event of ADV infection [16, 17], but this treatment has not received authorization from French regulatory agencies for this indication. Moreover, cidofovir is not devoid of side effects, especially renal toxicity. Efficacy can be limited when there is no concomitant antiviral immune reconstitution [5], and it is not easily available in some countries. A lipid-ester oral form of cidofovir, brincidofovir, is currently being evaluated for refractory ADV infection and disease in immunocompromised pediatric and adult patients [18–20]. A randomized placebo-controlled phase II study recently reported a clearance of ADV viral load using Brincidofovir (2 mg/kg if <50 kg, twice weekly) as a pre-emptive strategy in 67% patients compared to 33% in the placebo. However, results were impaired by gastrointestinal toxicity leading to early treatment discontinuation and more frequent incidence of acute graft versus host disease (GVHD) (50 vs 17%, respectively). Although the modulation of immunosuppression may be useful in controlling ADV infection after HSCT [21], it may however enhance the incidence and severity of GVHD. Thus, the development of other strategies is crucially required in the management of ADV infection after HSCT.

Adoptive transfer of ADV-specific T cells can restore specific antiviral immunity [21, 22]. Virus-specific T cells (VST) can be produced from the HSCT donor by cell culture during 2 to 8 weeks, or isolated by rapid immunomagnetic selection of IFN-γ-secreting T cells within less than 48 h [22–27]. Adoptive immunotherapy with VST was previously reported to be feasible and effective for the prophylaxis and treatment of EBV [23, 28, 29], CMV [30], ADV [22, 25, 26], and recently BKV infections [31]. Interferon-γ-based immunomagnetic isolation has the advantage of being a fast Good Manufacturing Practice (GMP)-grade procedure with a wide clinical implementation [22, 26, 31]. According to the literature, 123 patients (ADV *n* = 53; EBV *n* = 16; CMV *n* = 53, BKV *n* = 1) have been treated with a low dose of IFN-γ + VST (0.15 to 166 × 10^3^ VST/kg of patient body weight), leading to the reduction, or complete clearance, of viral load in 73.2% patients, a simultaneous in vivo expansion of VST and an acceptable tolerance profile [22, 25–30, 32–34]. Feuchtinger’s group reported in two studies infusion of IFN-γ + ADV-specific T cells from the HSCT donor into patients with chemo-refractory ADV infection/disease after MUD HSCT [22, 25]. The infusion of a low dose of adenovirus-specific T cell (ADV-VST) resulted in viral control and specific immune reconstitution without acute toxicity or significant onset of GVHD [22, 25]. However, no infusion of ADV-VST generated by IFN-γ-based immunomagnetic method has been reported so far in a third party haploidentical setting after UCB transplantation.

Based on the previous experience of our group concerning ADV and EBV-VST generation [35–37], we conducted a phase I/II multicenter pilot study consisting in an infusion of ADV-VST after HSCT in the event of refractory ADV infection or disease. Adenovirus-specific T cells were generated either from a (M)MUD or, for the first time with IFN-γ immunomagnetic method from a third party haploidentical donor for patients having undergone previous UCB transplantation. Specific anti-ADV immune reconstitution was observed in all patients, and viral load clearance in all but one.

## Methods

### Generation of clinical-grade ADV-specific T cells

Leukapheresis collections were performed in the initial HSC donors (*n* = 7) or related third party haploidentical donors (*n* = 6) for patients with UCB transplantation after informed consent. Briefly, recovered peripheral blood mononuclear cells (PBMCs) were stimulated for 6 h with a GMP adenovirus peptide pool: PepTivator-AdV5 Hexon (Miltenyi Biotec, Bergisch Gladbach, Germany). These cells were subsequently processed using the Cytokine Capture System (CCS, Miltenyi Biotec) based on an IFN-γ immunomagnetic technology on the CliniMACS device (Miltenyi Biotec) as previously described [35, 37]. Quality controls including microbiological seeding and flow cytometric assessment were performed on the positive fraction. When enrichment reached trial specifications (minimum lymphocyte viability of 20% and minimum enrichment of 15% in CD4+IFN-γ+ or CD8+IFN-γ+ T cells), freshly isolated ADV-specific T cells were released (*n* = 12) and brought to the different French investigating centers within 8 h following cell isolation. The maximum number of infused CD3+ cells was defined according to the SFGM-TC guidelines [38]: 5 × 10^4^ CD3/kg in a MUD setting, 1 × 10^4^ CD3/kg in a (M)MUD setting, and in a third party haploidentical setting after UCB transplantation.

### Phenotypic and functional controls

#### Phenotypic analysis

**Fig. 1:**
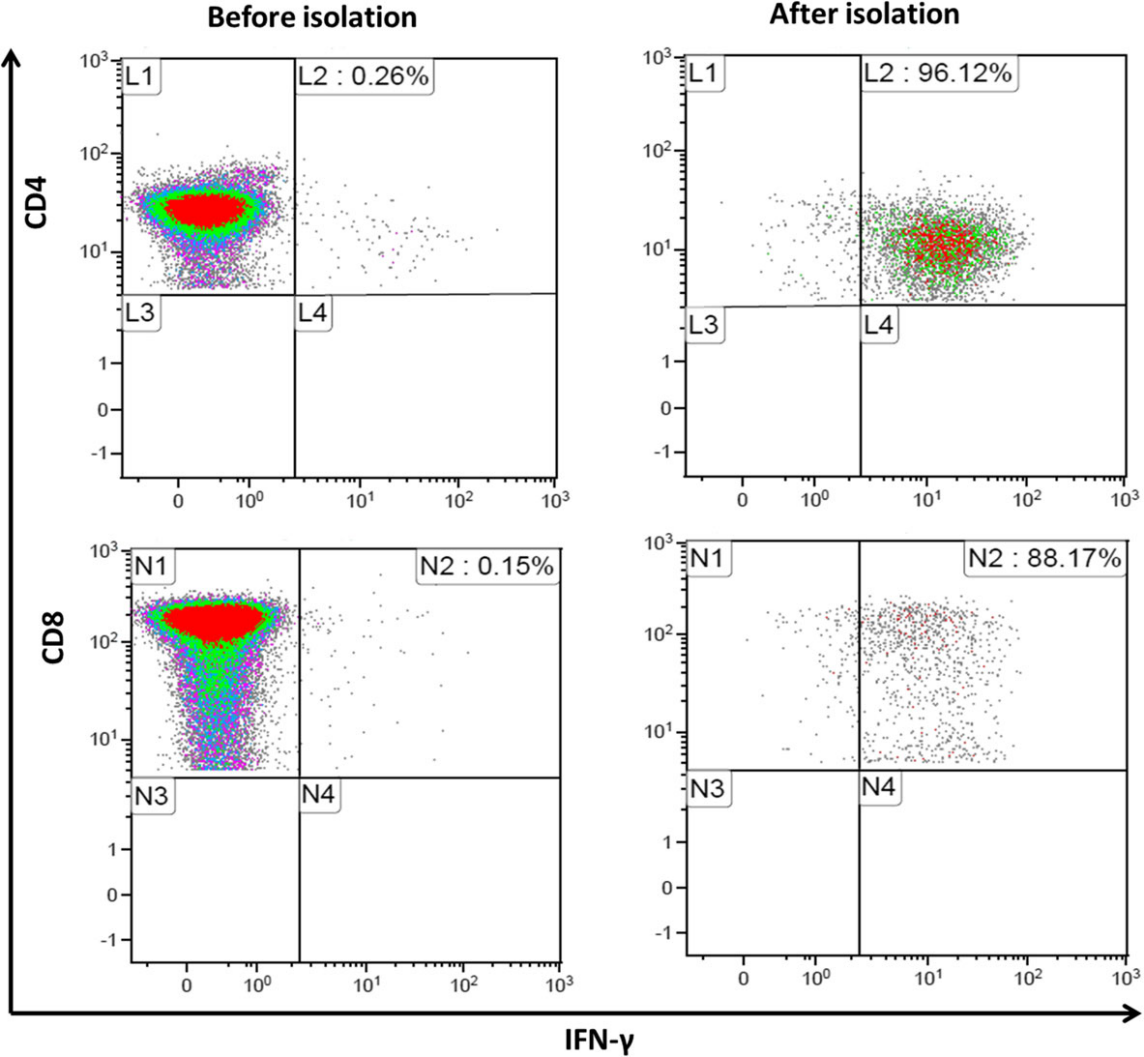
Representative dot plot of flow cytometry for ADV-VST. Enrichment of ADV-VSTs after IFN-γ-based immunomagnetic isolation compared with before isolation

As previously reported [35], frequency of PepTivator-AdV5 Hexon-specific T lymphocytes was assessed at different time points of the production process (before immunomagnetic selection, positive and negative fractions). At least 100,000 lymphoid cells were analyzed using Navios® cytometer (Beckman Coulter). Kaluza® software (v1.3; Beckman Coulter) was used for analysis of flow cytometry data. The percentage of IFN-γ-positive cells was analyzed in CD4+ and CD8+ T cells (Fig. 1).

#### In vitro functional controls

After in vitro expansion of a small sample of ADV-specific T cells for 2 to 4 weeks in the presence of IL-2 and irradiated autologous feeder cells from the negative fraction, enough cells were obtained to perform functional tests, as previously reported [35, 37]. Briefly, the capacity of expanded cells to secrete IFN-γ after PepTivator-AdV5 Hexon restimulation was controlled by IFN-γ Elispot assay and intra-cellular cytokine staining. A proliferative assay was performed with the DELFIA cell proliferation kit (PerkinElmer, Massachusetts, USA). Cytotoxicity assay was performed on productions of ADV-specific T cells, each time expansion resulted in sufficienT cells, to evaluate efficacy and absence of alloreactivity, respectively.

### Patients

#### General information

Fourteen patients, from nine different French HSCT centers, were eligible for the clinical trial IDRCB: 2010-A01029-30, between February 2012 and December 2014, and performed with the support of the French Society of Bone Marrow Transplantation and Cell Therapy (SFGM-TC). Inclusion criteria were (i) ADV infection or disease, refractory to antiviral drug therapy for at least 2 weeks or if an antiviral drug was not available for first-line therapy and (ii) acute GVHD ≤grade II controlled with two lines of immunosuppressive treatment or controlled chronic GVHD. This work was performed in accordance with Good Clinical Practices, approved by the local ethics committee and the French National Regulatory Agency (ANSM). Informed consent was obtained from all patients and/or their parents. The diagnosis of ADV infection was made on two consecutive positive ADV viremias [9]. Adenovirus disease was defined as ADV viremia associated with suggestive symptoms without there being any other obvious cause. A refractory adenovirus infection or disease was defined as an ADV viremia in which there was less than 0.5 Log decrease despite the antiviral therapy for at least 2 weeks. Table 1 presents patients’ characteristics. Follow-up was 180 days.

**Table 1:** Included patient characteristics

		MUD/MMUD	Unrelated UCB
		*N* = 6(5)*	*N* = 8(6)*
		*N*	*N*
Sex			
	Male	3 (2)	4 (2)
	Female	3	4
Age			
	<10	2	6 (4)
	10–20	2	1
	21–40	1	1
	>40	1 (0)	0
Diagnosis			
	ALL	2	1
	AML	0	2 (1)
	Hodgkin lymphoma	1	0
	Myelodysplastic syndrome	1	1
	Multiple myeloma	1 (0)	0
	Aplastic anemia	0	2 (1)
	Fanconi anemia	0	1
	Shwachman syndrome	1	0
	Primary immunodeficiency (HLA-II defect)	0	1
Graft			
	MUD	3 (2)	0
	MMUD	3	0
	Unrelated UCB	0	8 (6)
Conditioning regimen			
	Myeloablative	3	5 (4)
	Non myeloablative	3 (2)	3 (2)
T cell depletion			
	ATG	6 (5)	7 (5)
GVHD prophylaxis			
	Ciclosporin-A + MTX	3 (2)	0
	Ciclosporin-A + MMF	2	5 (4)
	Rapamycin + MMF	1	0
	MMF	0	1
	Ciclosporin-A + corticosteroid	0	2 (1)
GVHD status prior to ADV-VST transfer			
	No GVHD	1	4 (3)
	Acute GVHD grade I-II	5 (4)	1
	Acute GVHD grade III-IV	0	3 (2)
	Chronic GVHD	2	0
ADV status			
	Infection	1	2 (0)
	Disease	5 (4)	6
Antiviral treatment prior to ADV-VST transfer			
	Cidofovir	4 (3)	7 (5)
	Ribavirin	0	1
	None	2	0
Day ADV-VST transfer post-HSCT (days)			
	<50	0	2
	50–100	2	1
	>100	3	3
	Not infused	1	2

*(M)MUD* (mis-)matched unrelated donor, *UCB* umbilical cord blood, *ALL* acute lymphoblastic leukemia, *AML* acute myeloblastic leukemia, *ATG* antithymocyte globulin, *MTX* methotrexate, *MMF* mycophenolate mofetil, *GVHD* graft versus host disease, *ADV* adenovirus, *VSTs* virus-specific T cells

*Numbers of patients who received ADV-VST are presented in brackets

#### HSCT

All the patients, seven males and seven females (11 children and three adults), had previously undergone a HSCT for hematological malignancies (64.3%) or non-malignant disease (36.4%) including aplastic anemia, Fanconi anemia, Shwachman syndrome, and HLA-II defect. The source of hematopoietic stem cell was unrelated UCB in eight patients (57.1%) and peripheral hematopoietic stem cell in six patients (42.9%) including three HLA-matched (10/10 alleles, MUD) and three mismatched unrelated donors (9/10 alleles, MMUD). A myeloablative-conditioning regimen was performed in 57.1% of patients. All except one (02-08) received antithymocyte globulin (ATG) during the conditioning regimen. The main combinations of immunosuppressive drugs for GVHD prophylaxis were ciclosporin A-mycophenolate mofetil (50%) and ciclosporin A-methotrexate (21.5%).

After HSCT and before ADV-VST immunotherapy, GVHD occurred in most patients (9/14, 64.2%). Intensified immunosuppressive treatment was requested for all seven patients.

#### Adenovirus infection and disease

Asymptomatic ADV infection was observed in 21.4% of the patients (3/14) and ADV disease in 78.6%, predominantly in the gut (71.4%). Positive ADV viremia occurred after 100 days post-HSCT (50%), except in two patients (16.7%) including one who presented positive ADV viremia before HSCT. Prior to ADV-VST infusion, all the patients except two were treated with an antiviral drug (cidofovir (*n* = 8) or ribavirin (*n* = 1)) for at least 2 weeks. Patients were treated with an ADV-VST infusion after a mean of 52 ± 22 days from ADV infection diagnosis for the (M)MUD group and 45 ± 26 days for the UCB group (*p* = 0.64), including the shortest time of 16 days for the UCB-transplanted patient 12-14. Interestingly, in two patients (14.3%), adoptive immunotherapy by ADV-VST was administered as first-line treatment, because cidofovir was not available in France at that time.

#### Adenovirus infection and immune reconstitution monitoring

Adenovirus (ADV) load monitoring in the peripheral blood was centralized in the promoter center and performed by quantitative HAdV PCR with Adenovirus R-gene® assay kit (Argene, BioMerieux, Varilhes, France) before and after ADV-specific T cell infusion, twice a week for 3 weeks, every 2 weeks until month 3, and monthly until month 6. For patient 3, ADV load was negative in the blood but positive in stools although not quantified. Specific immune reconstitution was monitored by IFN-γ Elispot and proliferative assays at days 0, 14, 30, 60, and 90. The positive threshold in Elispot assay was previously defined at 72 SFC/10^6^ PBMC [35]. The reported results take into account ADV-specific IFN-γ secretion after removing the negative control (without stimulation) IFN-γ secretion.

### Statistical analysis

Statistical tests were performed using GraphPad Prism 5 software (San Diego, CA, USA). IFN-γ secretion of ADV-VST was analyzed using Student’s paired *t* test. In vivo IFN-γ immune response from D_14_ to D_60_ was compared with Wilcoxon’s signed-rank test; the other series were analyzed by the Mann Whitney test. Statistical significance was fixed a posteriori for a *p* value less than 0.05.

## Results

### Production of ADV-VST

Patient 04-09 was removed from the study because of the absence of ADV-specific response of the potential donor evaluated by IFN-γ Elispot assay and a concomitant clinical improvement. Production of ADV-VST was performed from peripheral blood mononuclear cells collected from the initial HSC donor for patients who were transplanted with (M)MUD (6 patients/13) or from a haploidentical third party donor for the 7 patients who were transplanted with UCB. A mean enrichment of 64.1 ± 32.0% CD4+IFN-γ+ T cells and 47.2 ± 34.2% CD8+IFN-γ+ T cells in CD4+ and CD8+ T cells, respectively, was obtained.

Absence of microbiologic contamination was attested. Functional tests showed that ADV-VST-expanded cells were still able to secrete IFN-γ (44,702 ± 20,266 SFCs/10^6^ cells versus 367 ± 160 SFCs/10^6^ PBMC; *p* < 0.02) and to proliferate (64,107 ± 62,563 cpm versus 32,794 ± 40,100 cpm; *p* = 0.074) after restimulation with PepTivator-AdV5 Hexon. In vitro efficacy of ADV-VST-expanded cells was attested for ten productions by a mean cytotoxicity of 24.75 ± 11.10% against PepTivator-AdV5 Hexon-pulsed autologous target cells, 1.60 ± 3.13% against non-pulsed autologous target cells and 2.89 ± 5.75% against non-pulsed allogeneic target cells (effector-to-target cell ratio = 10:1).

Two patients did not receive ADV-VST: one (patient 11-07) due to insufficient IFN-γ+ T cell enrichment according to trial specifications and one (11-02) due to logistic abnormality.

After ADV-VST enrichment, 11 patients received a mean dose of 5.05 ± 7.66 × 10^3^ CD4+IFN-γ+ T cells/kg (range 0.12 to 26 × 10^3^/kg) and 0.77 ± 0.65 × 10^3^ CD8+IFN-γ+ T cells/kg (range 0.04 to 2.10 × 10^3^/kg) (Table 2). No significant differences were observed between (M)MUD and haploidentical third party donor groups.

**Table 2:** Infused ADV-VST characteristics

Patient	ADV-VST origin	Donor ADV response (SFCs per 10^6^ PBMC)	VST dose (×10^3^ viables CD3+ IFNγ+/kg)	VST dose (×10^3^ CD4+ IFNγ+/kg) [enrichment (% of CD4)]	VST dose (×10^3^ CD8+ IFNγ+/kg) [enrichment (% of CD8)]
01-01	HSC donor	96	0.39	0.35 [68.4]	0.04 [7.1]
07-03	HSC donor	527	28.10	26.0 [78.5]	2.10 [15.8]
09-04	HSC donor	177	0.25	0.12 [51.0]	0.13 [45.9]
06-05	HSC donor	UN	0.92	0.34 [47.6]	0.58 [56.6]
07-06	Haplo donor (mother)	UN	1.21	0.66 [15.1]	0.55 [11.7]
02-08	Haplo donor (mother)	364	1.10	0.60 [87.7]	0.50 [76.4]
11-10	Haplo donor (sister)	446	3.90	2.99 [96.0]	0.90 [93.5]
12-11	Haplo donor (mother)	370	9.14	8.13 [94.3]	1.01 [90.6]
08-12	Haplo donor (mother)	561	8.28	7.31 [84.5]	0.98 [69.6]
01-13	HSC donor	271	1.41	1.34 [81.9]	0.08 [39.8]
12-14	Haplo donor (mother)	492	9.38	7.70 [96.1]	1.61 [88.2]
Mean		367	5.83	5.05 [72.8]	0.77 [54.1]
SD		160	8.23	7.66 [25.5]	0.65 [32.5]

*ADV-VST* adenovirus-specific T cells, *SFC* secretion-forming cells, *PBMC* peripheral blood mononuclear cells, *Haplo donor* haploidentical donor, *SD* standard deviation, *UN* unavailable

### ADV-VST infusion tolerance

ADV-VST infusion was immediately well tolerated with no adverse event, except one episode of chills without fever in one patient with spontaneous recovery.

Three patients experienced GVHD reactivation (27%) within the 30 days following the ADV-VST infusion. Among these three patients, one (06-05) presented extensive chronic GVHD at day 7 after ADV-VST infusion, whereas the other two presented grade I (07-06) or grade III (02-08) acute GVHD at D_14_. All these three patients developed a first episode of GVHD before the ADV-VST infusion. To note, patient 06-05 discontinued immunosuppressive drugs 1 month before the ADV-VST infusion to manage ADV reactivation. This patient received a dose of 920 CD3+IFN-γ+ cells/kg with a purity of CD4+ IFN-γ+ and CD8+IFN-γ+ T cells nearing 50%. Although cell enrichment was fair, allowing for contamination by non-specific and then potentially alloreactive T cells, the total CD3+ T cell dose infused remained up to five times lower than the maximum dose specified in the trial. In patient 02-08, immunosuppressive drugs were discontinued on the day of the ADV-VST infusion. This patient received 1100 CD3+ IFN-γ+ cells/kg with enrichment over 75% in CD4+ and CD8+IFN-γ+ T cells. However, acute GVHD reactivated in the days following ADV-VST infusion, requiring a further combination of different immunosuppressive drugs. Concerning patient 07-06, immunosuppression, consisting of combined mycophenolate mofetil and corticosteroids, was implemented at day 70 post-HSCT for grade II acute GVHD and was maintained during and after ADV-VST infusion; although, GVHD was controlled. At D_14_ post ADV-VST infusion, reactivation of acute GVHD did not require increased immunosuppression.

Besides, more than 1 month after ADV-VST infusion, two other patients experienced GVHD during follow-up. One patient (01-01) presented with moderate chronic GVHD reactivation at D_45_ post-infusion and then extensive chronic GVHD at 6 months, after immunosuppressive drug tapering. The second patient (12-14) presented with de novo grade II acute GVHD at 3 months post ADV-VST infusion. Although a causal link with ADV-VST infusion cannot be excluded for the two patients with late-onset GVHD, GVHD was not ascribed to adoptive immunotherapy according to the study protocol.

Among the five patients with GVHD reactivation after infusion, two received ADV-VST from (M)MUD (1 MUD with late GVHD and 1 MMUD with early GVHD) and three from a third party haploidentical donor (two early GVHD and one late GVHD).

In the context of third party haploidentical cell origin, compatibility between ADV-VST and UCB graft concerned 2/6 HLA loci, to 6/6 among HLA-A, HLA-B, and HLA-DRB1, including three patients with HLA semi-identity (Table 3).

**Table 3:** Compatibility between ADV-VST and UCB onto HLA-A, HLA-B, and HLA-DRB1

Patient	UCB1	UCB2
07-06	Semi-identical	
02-08	Matched 6/6	4/6 with MM HLA-A
11-10	2/6 with MM HLA-A	
12-11	Semi-identical	
08-12	2/6 with MM HLA-B	
12-14	Semi-identical	

One DRB-1 compatibility was always observed

*UCB* umbilical cord blood, *VSTs* virus-specific T cells, *MM* mis-matched

### Immune reconstitution

**Fig. 2:**
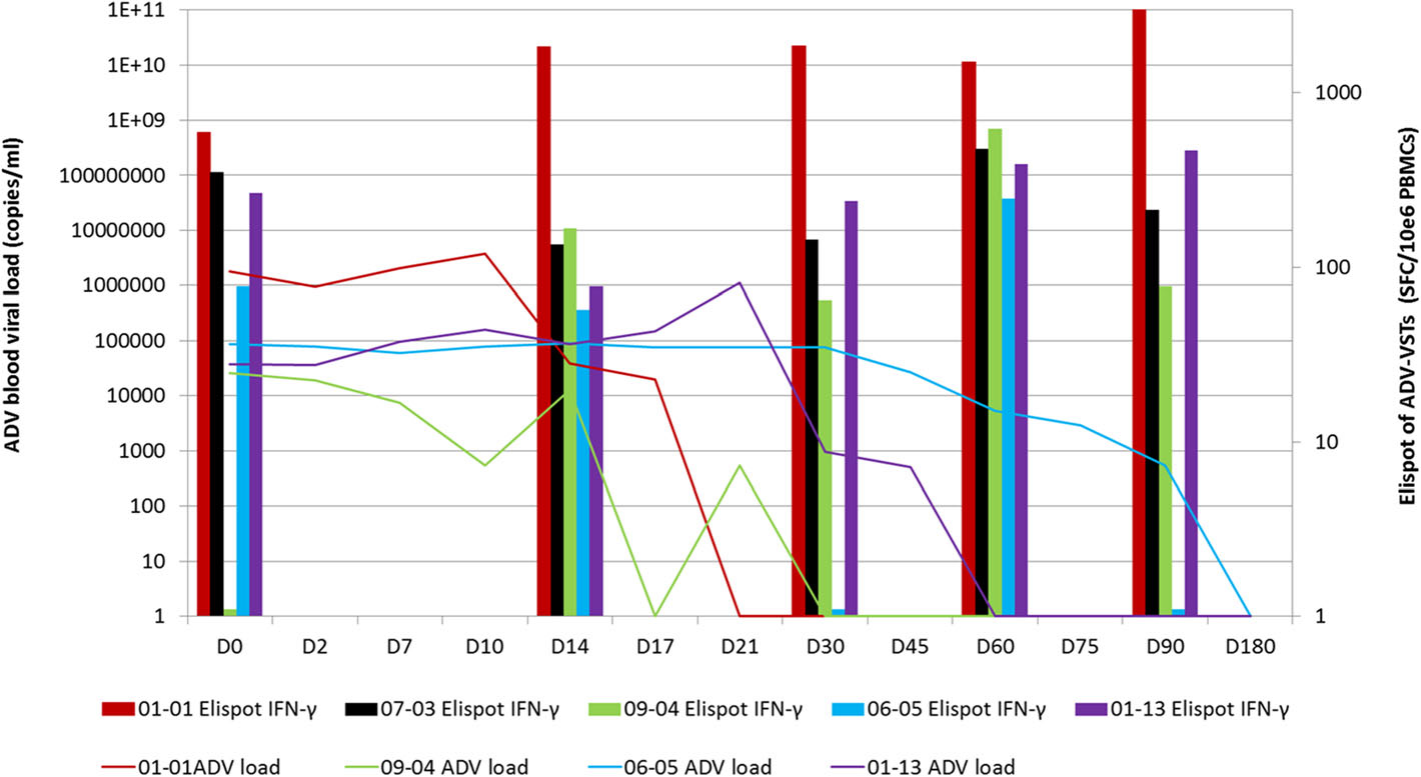
Evolution of ADV viral load and specific immune reconstitution after (M)MUD ADV-VST infusion. Five patients who received a (M)MUD ADV-VST infusion presented ADV immune response at D_14_, the highest IFN-γ immune response was mainly observed at D_60_ (*column with right y-axis value*). Clearance of ADV viral load (*line with left y-axis value*) in the peripheral blood was observed in four patients; patient 07-03 had no ADV viral load evaluable in blood but in stools without quantification and cleared at D_21_

**Fig. 3:**
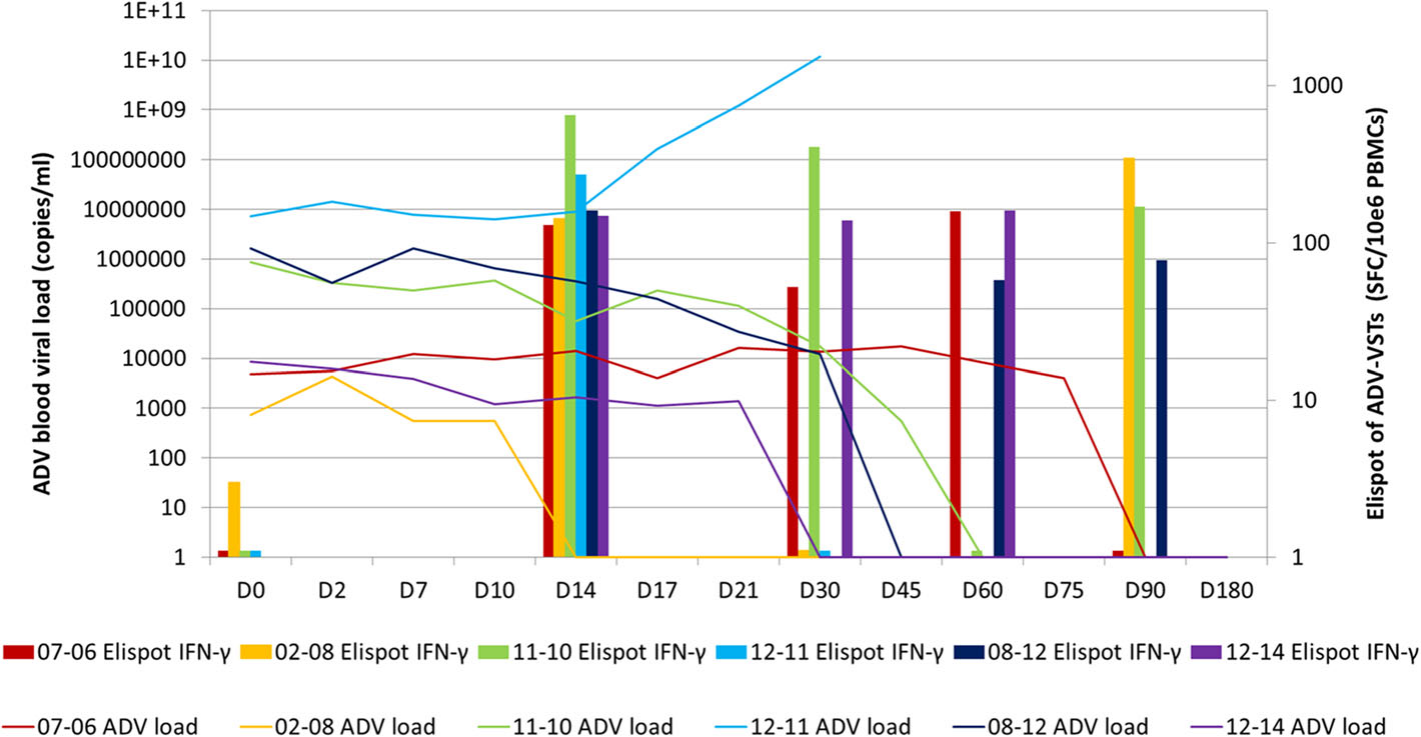
Evolution of ADV viral load and specific immune reconstitution after haploidentical related ADV-VST infusion. Six patients who received a third party haploidentical donor ADV-VST infusion after UCB transplantation presented ADV immune response at D_14_ (*column with right y-axis value*). Clearance of ADV viral load (*line with left y-axis value*) in peripheral blood was observed in five patients; patient 12-11 was stabilized until D_17_ and highly increased at D_30_ until death due to multisystemic adenovirus disease

We analyzed ADV-specific immune reconstitution by IFN-γ Elispot assay. After infusion, in vivo expansion of ADV-VST was detected in all patients according to variable kinetics. Among the five patients who received a (M)MUD ADV-VST infusion (Fig. 2), four (80%) showed an IFN-γ response above the positive threshold at D_0_, whilst this was not observed in any patient receiving haploidentical ADV-VST after UCB transplantation (Fig. 3). Indeed, patient 01-01, who already had a high ADV immune response at D_0_, experienced a huge expansion of ADV-VST which was maintained at D_90_, probably by an added effect of ADV-VST. Complete clearance of ADV viral load was observed at D_21_ (Fig. 2).

The ADV immune response was always positive at D_14_ (209 ± 171 SFC/10^6^ PBMC), except for patient 06-05 (57 SFC/10^6^ PBMC). A tendency to a decrease in IFN-γ immune response was usually observed at D_30_ (131 ± 138 SFC/10^6^ PBMC; *p* = 0.15) whilst the highest IFN-γ immune response was mostly observed at D_60_ (304 ± 204 SFC/10^6^ PBMC; *p* = 0.063). Two patients presented a fluctuating immune response, while a favorable evolution of ADV viral load was finally observed. We did not see any difference between the (M)MUD and UCB groups regarding intensity and persistence of ADV-specific immune response (Figs. 2 and 3).

Regarding patient 12-11, ADV-VST expansion was detected at D_14_ but not at D_30_, while ADV viral load, which was the highest at the time of ADV-VST infusion (7.3 × 10^6^ copies/ml), dramatically increased (Fig. 3).

### ADV response

ADV viral load was monitored by PCR in the peripheral blood and was also positive for patients with gastrointestinal disease. Due to an isolated gastrointestinal ADV disease, patient 07-03 did not have ADV evaluable viral load in the blood but in the stools (without quantification) and which cleared at D_21_. ADV viral load in the peripheral blood cleared in nine out of ten patients (90%). Clearance of ADV viral load was achieved at D_60_ in seven (63.6%) patients (3/5 patients with (M)MUD (Fig. 2) and 4/5 patients with third party haploidentical donor (Fig. 3)) including one with the earliest clearance at D_14_ (patient 02-08). Clearance was achieved later in two other patients, at D_90_ (patient 07-06, third party haploidentical donor) and at D_180_ (patient 06-05, (M)MUD). Patient 07-06 presented with a persistent viral load (over 1 × 10^4^ copies/ml) until D_45_ which decreased until clearance at D_90_. For patient 06-05, the ADV viral load remained stable until D_30_, with a mild in vivo expansion of ADV-VST concomitant with a decreased ADV load until clearance at D_180_. Considering patient 12-11, the viral load was the highest of all patients, 7.3 × 10^6^ copies/ml at D_0_ of ADV-VST infusion. The ADV viral load was stabilized during the first 2 weeks post-infusion, but a dramatic increase in the ADV viral load occurred from D_17_ (1.63 × 10^8^ copies/mL) to D_30_ (11.63 × 10^9^ copies/mL) leading to death at day 33 due to multisystemic ADV disease.

Finally, patients receiving ADV-VST either from a (M)MUD or a third party haploidentical donor showed specific ADV-VST expansion after infusion, except for one patient who received third party haploidentical ADV-VST and experienced fatal ADV disease (Table 4).

**Table 4:** ADV and GVHD status before and after ADV-VST infusion for each patient

Patient	Diagnosis	Age	AlloSCT	GVHD status at inclusion	Early GVHD after VSTs (<1 month)	Days of ADV reactivation Since graft	D0 ADV load (×10^3^ copy/mL)	ADV status	Modulation of IS for ADV management	IS at VSTs infusion	Antiviral treatment prior VSTs	Days of VSTs since ADV reactivation	Antiviral treatment after VSTs	ADV load response (decrease > 0.5log)	ADV IR	Early infection (<1 month)	Outcome (ADV clearance)
01-01	ALL	14	MAC-ATG PHSC MUD	Grade II aGVHD	No	100	1790	Disease (GT)	None	Cyclo + CST + basiliximab	Cidofovir	41	Cidofovir D0-D9	D14	Increase at D14	EBV at D21	Dead > D180 (D21)
07-03	MDS	2	RIC-ATG PHSC MUD	Grade I aGVHD	No	143	NA	Disease (GT)	None	MMF	Cidofovir	78	Cidofovir D11-D24	UN	Increase at D60	No	Alive (D21)
09-04	ALL	14	MAC-ATG PHSC MMUD	Grade II aGVHD + cGVHD	No	70	25.8	Asymptomatic infection	Stop IS for ADV, restart at D0	Cyclo + CST	None	23	None	D17	Increase at D14	EBV at D14	Alive (D30)
06-05	HL	31	RIC-ATG PHSC MMUD	Grade II aGVHD + limited cGVHD	Extensive cGVHD (D7)	215	87.1	Disease (GT)	Stop IS for ADV, restart at D32	None	None	49	None	D45	Increase at D60	No	Alive (D180)
07-06	AA	12	RIC-ATG UCB	Grade II aGVHD	Grade I aGVHD (D14)	46	4.82	Disease (GT)	None	MMF + CST	Ribavirine	95	Ribavirine D0-D40 + Cidofovir D60-D115	D60	Increase at D14	No	Dead > D180 (D90)
02-08	ALL	5	MAC UCB	Grade III aGVHD	Grade III aGHVD (D14)	64	0.75	Disease (pulmonary)	Stop IS for VSTs and restart at D12	None	Cidofovir	42	None	D7	Increase at D14	Bacteria at D7	Dead of infection at D132 (D14)
11-10	MDS	27	MAC-ATG UCB	Grade IV aGVHD	No	126	877	Disease (GT)	None	Cyclo + CST + Ruxolitinib	Cidofovir	37	None	D21	Increase at D14	Bacteria at D21	Alive (D60)
12-11	AML	11 m	MAC-ATG UCB	No GVHD	No	18	7300	Disease (GT)	Stop MMF for ADV	Cyclo	Cidofovir	43	Ribavirine until D8	No response	Increase at D14, noresponse at D30	EBV at D14	Dead of ADV at D33
08-12	PID	7 m	MAC-ATG UCB	No GVHD	No	-14	1660	Disease (GT)	Stop CST for ADV	Cyclo	Cidofovir	37	None	D10	response at D14	Bacteria at D7 and D21	Alive (D45)
01-13	SS	8	MAC-ATG PHSC MMUD	No GVHD	No	19	37.9	Disease (GT)	Stop IS for ADV	None	Cidofovir	69	Cidofovir D6-D23	D30	Increase at D30	No	Alive (D60)
12-14	FA	4	RIC-ATG UCB	No GVHD	No	13	8.5	Disease (GT)	None	Cyclo + MMF Stop MMF D4	Cidofovir	16	Brincidofovir D95-D125 + Cidofovir D125-D132	D70	Response at D14	No	Alive (D30)

All the patients who received UCB transplantation were infused with ADV-VST isolated from third party haplo-identical donors. All the patients who received PHSC transplantation were infused with ADV-VST isolated from original HSCT donors

*MAC* myeloablative conditioning, *ATG* antithymocyte globulin, *RIC* reduce-intensity conditioning, *PHSC* peripheral hematopoietic stem cells, *(M)MUD* (mis-)matched unrelated donor, *phenol UCB* phenoidentical umbilical cord blood, *ALL* acute lymphoblastic leukemia, *AML* acute myeloblastic leukemia, *MDS* myelodysplastic syndrome, *HL* Hodgkin lymphoma, *AA* aplastic anemia, *PID* primary immunodeficiency, *SS* Shwachman syndrome, *FA* Fanconi anemia, *IS* immunosuppressives drugs, *Cyclo* cyclosporine, *CST* corticosteroid, *MTX* methotrexate, *MMF* mycophenolate mofetil, *ECP* extracorporal photopheresis, *a/cGVHD* acute/chronic graft versus host disease, *ADV* adenovirus, *VSTs* virus-specific T cells, *IR* immune reconstitution, *D*_*0*_ day of VSTs infusion, *m* month, *GT* gastrointestinal tract, *UN* unavailable, *NA* not applicable

i. Antiviral drug treatments
  Among the nine patients whose ADV infection was treated with antiviral drugs before ADV-VST infusion, three continued treatment after ADV-VST infusion (33.3%), two for a short time (less than 10 days for patients 01-01 and 12-11) probably whilst waiting for ADV-VST efficacy and one of them for 4 months (patient 07-06) because of a persistent viral load. Antiviral therapy was restarted soon after ADV-VST infusion and continued for 2 weeks, for patients 07-03 and 01-13, because of increased viral load during the first week post-infusion or because of persistent clinical symptoms of ADV disease. Patient 12-14 received brincidofovir at D_95_ post-infusion (twice weekly for 3 weeks) followed by cidofovir until D_132_. This was for ADV reactivation in stools without any virus being detected in the blood, concomitant to digestive GVHD and intensified immunosuppression. In patient 12-11, ADV viral load did not decrease.
  Five patients (45.5%) did not receive any anti-ADV drugs after ADV-VST infusion, including the two patients whose ADV infection (*n* = 1) or disease (*n* = 1) were preemptively treated only by ADV-VST infusion. All five patients experienced ADV viral load clearance.
ii. Modulation of immunosuppression
  A modulation of immunosuppression was performed for seven patients, five for ADV disease and two for ADV-VST infusion. Immunosuppression was definitely stopped (*n* = 1) or discontinued up to 1 month before ADV-VST infusion (*n* = 2) or at the time of ADV-VST infusion (*n* = 1) until D_12_. It was reduced in three patients. Among the seven patients, a positive IFN-γ immune response was detected at D_14_ for five of them and later (D_30_ and D_60_) for the last two.
  At the time of ADV-VST infusion, four patients received corticosteroids (1 or 2 mg/kg) in combination with ciclosporin-A and/or MMF, while a positive IFN-γ immune response was detected at D_14_ in all of them. However, in vivo ADV-VST expansion fluctuated for patient 07-06, whilst immunosuppression was increased (ECP sessions followed by rapamycine). Conversely, the second patient (06-05), who also presented a fluctuation in ADV-VST expansion, received PUVA therapy exclusively from D_32_. As previously mentioned, all patients except one (02-08) received ATG during the conditioning regimen with a median time between HSCT and ADV-VST infusion of 117 days.
  In our cohort, we did not see any impact of the type of immunosuppression on in vivo ADV-VST expansion, regardless of (M)MUD or UCB HSCT.

### Outcome

Eleven patients received ADV-VST infusion, one for asymptomatic infection (09-04), one for pulmonary ADV disease (02-08), and nine for digestive ADV disease. Ten patients experienced a decreased viral load and nine a complete resolution of ADV symptoms including pulmonary disease, five without any other antiviral treatment. Patient 12-14 experienced digestive ADV reactivation at D_95_ requiring antiviral drug therapy. Patient 09-04 was preemptively treated with ADV-VST with rapid clearance of ADV viral load.

During the 180 day follow-up post-ADV-VST infusion, two patients died. Patient 02-08 died at D_132_ from sepsis secondary to bacterial infection in the context of uncontrolled acute GVHD whilst the ADV disease was resolved. Patient 12-11 died at D_33_ of resistant multisystemic adenovirus disease. Adenovirus viral load was first stabilized with expanded ADV-VST, but increased again; although, no implementation of immunosuppressive treatment occurred.

Moreover, two patients (01-01 and 07-06) died more than 6 months post-ADV-VST infusion at D_191_ and D_209_, respectively, due to multi-organ failure secondary to non-viral infections in the context of acute GVHD reactivation. Nine patients were alive (81.8%) at the end of the 6-month follow-up and seven (63.6%) at the end of the study with a follow-up of at least 18 months and up to 42 months.

## Discussion

Adenovirus infections and reactivations after HSCT are difficult to manage without specific immune reconstitution, even when effective antiviral drugs are available. Thus, a promising way of managing these infections relies on a restored specific immunity as observed after ADV-VST infusion.

We proposed to evaluate the feasibility, tolerance, and efficacy of an ADV adoptive immunotherapy by the infusion of ADV-VST enriched thanks to an immunomagnetic isolation method based on IFN-γ secretion after in vitro exposure to ADV antigens. We focused on a cohort of patients who received either (M)MUD or UCB HSCT. To our knowledge, this is the first such report of six UCB-transplanted patients receiving adoptive IFN-γ-selected immunotherapy from a third party haploidentical donor. ADV-VST infusion was usually performed in case of anti-ADV drug treatment failure or toxicity but, interestingly, two patients were efficiently preemptively treated with ADV-VST alone.

Considering the 13 ADV-VST preparations, enrichment using the Cytokine Capture System on the CliniMACS device (Miltenyi Biotec) was successfully achieved except in one case where it was lower than 15%. The low proportion of ADV-VST in the donor peripheral blood was attested by Elispot assay; although, frequency of ADV-VST detected in flow cytometry before selection was similar to other donors. However, mean fluorescence intensity seemed low in this case. Actually, immunomagnetic isolation requires consistent IFN-γ secretion to target and capture specific cells, attested by the minimum size defined for the counted spots.

In a related haploidentical setting and if there is more than one possible related donor, screening of the optimal donor may be useful. We performed such screening for all the following inclusions. Accessibility to a third party haploidentical donor is usually easier compared to a third-party-unrelated donor or a (M)MUD donor, as donor centers require time to pronounce the ethics committee’s decision to release the donor. This shorter time lapse (although not significant in our study) is critical in a pre-emptive transfer setting of ADV-VST.

During follow-up, five patients presented GVHD with a total of 13 episodes. Three patients presented early onset of GVHD reactivation in the first month after ADV-VST infusion, one patient received ADV-VST generated from his previous MMUD, and two received it from third party haploidentical donors, whereas no de novo GVHD was observed. There was no difference in early onset of GVHD between (M)MUD- and haploidentical-donor-derived ADV-VST. In patient 06-05, considering the short time to GVHD reactivation [39], the low dose of infused T cells, the pathophysiology of chronic GVHD [40], and corticosteroid discontinuation 1 month before ADV-VST infusion, the reactivation cannot be attributed clearly to immunomodulation or to ADV-VST infusion. Likewise, immunosuppressive drugs were rapidly tapered in patient 02-08 aiming to manage the ADV infection whilst waiting for ADV-VST infusion. The grade III acute GVHD reactivation cannot be easily attributed to the discontinuation of immunosuppressive drugs on the day of ADV-VST infusion or to ADV-VST infusion itself. Although GVHD occurring after the first month post-infusion was not considered as an adverse event of ADV-VST infusion in the clinical trial, we reported a GVHD reactivation in patient 01-01 which worsened and became an extensive chronic GVHD after the sudden cessation of immunosuppressive drugs by the patient himself. We also reported a de novo acute grade II GVHD in patient 12-14 at day 90 following ADV-VST infusion and occurring 28 days after UCB transplantation. These cases highlight the difficulty of precisely determining the cause of GVHD reactivation, or its worsening, in the first month after ADV-VST infusion and of de novo GVHD occurrence later.

Despite a very low cell dose of infused ADV-VST (5830 ± 8230 CD3+ IFN-γ + T cells/kg, Table 2), patients presented ADV-specific immune reconstitution in the following 2 weeks, still detectable after 3 months, except for patient 12-11. Interestingly, patient 09-04 preemptively received 250 CD3+ IFN-γ+ T cells/kg and presented a clearance of ADV viral load with an in vivo expansion of ADV-VST. Similarly, Feucht et al. reported that a dose as low as 312 ADV-specific CD3+ T cells/kg showed effective in vivo expansion with clearance of ADV viremia [22]. In the context of haploidentical ADV-VST infused after UCB transplantation, and despite a variable compatibility between 2/6 with biallelic HLA class I (−A or −B) mismatched and 6/6 (patient 02-08), efficacy of ADV-VST was not impaired probably because at least one HLA class I and class II compatibilities were always preserved, as reported in the context of EBV-VST banking [26, 41–43]. No toxicity against hematopoietic stem cell graft was observed.

One difference observed between the two groups (UCB and (M)MUD transplantation) was the presence of an ADV-specific immune reconstitution at D_0_ for most of (M)MUD-transplanted patients, while this was never the case in the UCB transplanted group probably due to delayed immune reconstitution after UCB transplantation [44, 45] and earlier infusion of ADV-VST compared to the (M)MUD-transplanted group. As observed with the dramatic increase in specific immune response in patient 01-01, further expansion of ADV-VST was not impaired by this pre-existing immunity. We cannot conclude whether this immune reconstitution prior ADV-VST infusion could be enough to allow ADV clearance.

In ten out of 11 patients, in vivo expansion of ADV-VST was associated with a clearance of ADV viral load either in blood or in stools. Among six patients receiving antiviral drugs after ADV-VST infusion, five presented concomitant in vivo expansion of ADV-VST with successful ADV viral load clearance. Moreover, five patients who did not receive any antiviral drugs after ADV-VST infusion also presented effective ADV viral load clearance, including the two patients whose ADV infections were preemptively treated only by ADV-VST infusion. Although the number of patients was small, this last result is promising and suggests a correlation between ADV-VST expansion and ADV viral clearance, regardless of antiviral drug administration, as previously reported [22, 33, 34]. In one case, patient 12-11, ADV viral load was not controlled by combined antiviral drug and ADV-VST; although, an in vivo expansion of ADV-VST was observed leading to a transient balance between ADV viral load and ADV-VST (Fig. 3). ADV viral load was probably too high at the time of ADV-VST infusion, which did not have enough time for in vivo expansion and control of ADV replication. As UCB hematopoietic stem cell and ADV-VST were semi-compatible, no defect in ADV antigen presentation by donor’s dendritic cells could be incriminated, as attested by the first in vivo expansion of ADV-VST. Among evaluable patients, the number of mature effector memory T cell subsets (T_EM_), responsible for the immediate cytotoxic effect of ADV-VST but with a limited expansion capacity [37], was the highest in ADV-VST of patient 12-11 (T_EM_ 8.97 × 10^3^ vs 3.3 ± 3.36 × 10^3^/kg, range from 0.19 × 10^3^ to 8.34 × 10^3^/kg, Additional file 1). However, immature memory T cell subsets (T_CM_ and T_SCM_), responsible for the sustained in vivo efficacy of ADV-VST and with a high expansion potential [37], were the lowest for this patient (T_SCM_ 0/kg vs 49 ± 42/kg; T_CM_ 56/kg vs 145 ± 69/kg). This could explain the absence of a persistent response and the delayed differentiation and expansion from those small immature compartments in a short time in patient 12-11. In vivo ADV-VST expansion is crucial as low doses of ADV-VST were infused in this cohort, but requires time.

Such a sequential action of the different T cell compartments is supported by the evolution (from D_14_ to D_60_) of the IFN-γ+ immune response after ADV-VST infusion in the whole cohort.

We noted that maintaining immunosuppression after ADV-VST infusion, with ciclosporin-A or MMF possibly combined with corticosteroids, impaired neither in vivo ADV-VST expansion nor viral clearance. Similarly, other groups have reported that in vivo ADV-VST expansion with cleared viremia was observed, despite the immunosuppression after ADV-VST infusion [22, 26, 46]. Ten out of eleven patients received ATG as part of the conditioning regimen with a median time between HSCT and ADV-VST infusion of 117 days (range, 23 to 264 days). Unlike with Feucht et al, nine presented in vivo ADV-VST expansion and clearance of ADV viral load [22]. We suggest that, in our study, ATG no longer influenced the in vivo expansion of ADV-VST at transfer.

Ljungman et al. reported an ADV-related mortality rate ranging from 8 to 54%, with higher rates in patients with ADV pneumonia (73%) and disseminated disease (61%) [47]. In our eleven patients with refractory ADV infection (*n* = 1) or disease (*n* = 10, including one ADV pneumonia) after HSCT, one ADV-related death was reported (9%). Three other patients died from non-viral infections in the context of GVHD reactivation. Although the sample size is small in our cohort, the encouraging results demonstrated that adoptive ADV-VST immunotherapy could obviously improve survival rate for patients with refractory infection or disease after HSCT.

## Conclusions

This phase I/II multicenter clinical trial presented encouraging evidence of the efficacy of adoptive ADV-VST immunotherapy after HSCT for hematological malignant or non-malignant diseases. Although some cases of GVHD occurred during the first month after ADV-VST, it is impossible to discriminate between the accountability of ADV-VST themselves and modulation of immunosuppressive drugs in those patients waiting for ADV-VST infusion. This therapeutic approach is fast and can be implemented in advanced therapy medicinal product laboratories for widespread clinical application. We reported interesting results in UCB-transplanted patients using a third party haploidentical donor. This opens new horizons for the treatment of infections in those patients whose HSCT donor is no longer available. Fresh leukapheresis for ADV-VST production can be arranged quickly from a third party haploidentical donor, while it is more complicated from a third party-unrelated donor. Moreover, we observed efficacy in the two preemptively treated patients without any other antiviral drug, raising hopes that ADV-VST can be used as first-line treatment. This will help to treat patients with lower ADV viral loads, letting time for expansion of infused ADV-VST. We now need a randomized, controlled study in a large cohort of patients comparing antiviral treatment alone to antiviral treatment combined with ADV-VST in order to confirm safety and efficacy.

## Additional file

Additional file 1: Online supplement. (DOCX 21 kb)

## Abbreviations

(M)-MUD: Mis-matched or matched unrelated donors
ADV: Adenovirus
ADV-VST: Adenovirus-specific T cell
ATG: Antithymocyte globulin
CMV: Cytomegalovirus
EBV: Epstein-Barr virus
GVHD: Graft versus host disease
HLA: Human leukocyte antigen
HSCT: Hematopoietic stem cell transplantation
IFN-γ: Interferon-γ
PBMC: Peripheral blood mononuclear cell
PTLD: Post-transplant lymphoproliferative disease
SFC: Spot-forming cell
T_CM_: Central memory T cell
T_EM_: Effector memory T cell
T_SCM_: Stem memory T cell
UCB: Umbilical cord blood
VST: Virus-specific T cell
