# Supplementary material for: Curative or pre-emptive adenovirus-specific T cell transfer from matched unrelated or third party haploidentical donors after HSCT, including UCB transplantations: a successful phase I/II multicenter clinical trial
Source: J Hematol Oncol. 2017 May 8;10:102. doi: 10.1186/s13045-017-0469-0 (PMC5421327; doi:10.1186/s13045-017-0469-0)
Supplement: Additional file 1: — Online supplement. (DOCX 21 kb) [file 13045_2017_469_MOESM1_ESM.docx]

**Online supplement**

**Methods**

***Intracellular staining and sub-populations analysis***

Intracellular staining was performed before and after immunomagnetic selection to detect the IFN-γ secretion capacity and analyze different sub-populations in ADV-VST according to our previous report [[1](#_ENREF_1)]. Staining of viable cells was performed using LIVE/DEAD AQUA fluorescent-reactive dye (Life Technologies, Saint Aubin, France), anti-CD3, -CD4, -CD8, -CD45RA (BD Biosciences, New Jersey, USA), -CD197 (CCR7) (BD Biosciences), -CD95 (Biolegend, San Diego, CA, USA) and -IFN-γ antibodies. CCR7 and CD45RA expressions were used to differentiate four sub-populations of CD3+CD4+IFN-γ+ and CD3+CD8+IFN-γ+ ADV-VST: T naive-like cells (T_N-like_) were defined as CD45RA^+^CCR7^+^; central memory T-cells (T_CM_) as CD45RA^-^CCR7^+^; effector memory T-cells (T_EM_) as CD45RA^-^CCR7^-^ and effector T-cells (T_EFF_) as CD45RA^+^CCR7^-^. Finally, CD95 staining was used to discriminate between naive T-cells (T_N_) (CD45RA^+^CCR7^+^CD95^-^) and T memory stem cells (T_SCM_) (CD45RA^+^CCR7^+^CD95^+^), two subsets of T_N-like_. These analyses were performed by flow cytometry using the Navios® cytometer (Beckman Coulter). Kaluza® software (v1.3; Beckman Coulter) was used for analysis of flow cytometry data. The mean number of events acquired in the CD3 IFN-γ gate was 160±133 before isolation and 3345±3214 after isolation.

**Results**

***Production of ADV-VST***

Eleven of 13 donors were evaluable for the response to PepTivator-AdV5 Hexon by IFN-γ Elispot assay at the time of immunomagnetic selection (312 ± 188 SFCs/10^6^ PBMC, range 31 from 561 SFCs/10^6^ PBMC). For UCB transplanted patients, in 5 cases, haploidentical donors could be tested beforehand for ADV-specific IFN-γ response and the best responder was chosen for ADV-VST isolation (447 ± 83 SFCs/10^6^ PBMC). In comparison, ADV-specific IFN-γ secretion in the 5 evaluable (M)MUD was lower (234 ± 178, p=0.10).

***General immune recovery of after ADV-VST infusion***

Lymphocyte, CD3+, CD4+ and CD8+ T cell recovery was detected after ADV-VST infusion. A slight increase in general immune recovery was observed at D14 and was amplified after D30 (Figure S1).

***Outcome:***

During follow-up, we observed 30 infectious episodes (other than ADV) in 10 patients with 7 early infectious complications (within the first month post ADV-VST infusion). Bacterial infections were the most frequent (50%) and CMV- and EBV-reactivations post ADV-VST infusion represented 17% of the infections. Infectious complications evolved favorably, except for patient 02-08.

**References**

1. Qian C, Wang Y, Cai H, Laroye C, De Carvalho Bittencourt M, Clement L, Stoltz JF, Decot V, Reppel L, Bensoussan D: **Adenovirus-specific T-cell Subsets in Human Peripheral Blood and After IFN-gamma Immunomagnetic Selection.** *J Immunother* 2016, **39:**27-35.

**Legend**

**Figure S1: General immune recovery of after ADV-VST infusion.** A slight increase in general immune recovery was observed at D_14_ and was amplified after D_30_.
